# Case Report: Dose-dependent response to oclacitinib in a dog with Sézary syndrome

**DOI:** 10.3389/fvets.2025.1645059

**Published:** 2025-12-15

**Authors:** Jungmin Lym, Yeon Chae, Sanggu Kim, Sungjae Lee, Yoonhoi Koo, Hakhyun Kim, Byeong-Teck Kang, Soochong Kim, Taesik Yun

**Affiliations:** 1 Laboratory of Veterinary Internal Medicine, College of Veterinary Medicine, Chungbuk National University, Cheongju, Chungbuk, Republic of Korea; 2 Laboratory of Veterinary Pathology and Platelet Signaling, College of Veterinary Medicine, Chungbuk National University, Cheongju, Chungbuk, Republic of Korea; 3 Laboratory of Veterinary Internal Medicine, College of Veterinary Medicine, Kyungpook National University, Daegu, Republic of Korea

**Keywords:** cutaneous epitheliotropic T-cell lymphoma, dog, Janus kinase, JAK inhibitor, oclacitinib, Sézary syndrome, skin tumor

## Abstract

Cutaneous epitheliotropic T-cell lymphoma (CETL) is a rare malignant canine skin tumor. Sézary syndrome, a rare and aggressive subtype of CETL, lacks established treatment guidelines in veterinary medicine. A 10-year-old, spayed female Yorkshire Terrier presented with multiple skin nodules, plaques, crusts, and pruritus. Histopathological and immunohistochemical evaluations confirmed CETL, and Sézary cells were identified in a peripheral blood smear, confirming Sézary syndrome. The dog was initially treated with oclacitinib 0.7 mg/kg twice daily. Marked clinical improvement was observed on day 15 with the disappearance of Sézary cells. However, by day 32, the skin lesions had worsened, and the oclacitinib dose was increased to 3 mg/kg twice daily. Subsequent improvements were noted within 12 days, although a relapse occurred on day 63. Immunohistochemical staining revealed moderate and diffuse cytoplasmic and nuclear expression of Janus kinase 1 (JAK1), an oclacitinib target. This case demonstrated that both low- and high-dose oclacitinib may offer temporary clinical benefits in dogs with Sézary syndrome, potentially via JAK1 inhibition. Although the duration of the response was limited, the survival period exceeded that of previously reported canine cases of Sézary syndrome. These findings support further investigation of Janus kinase inhibitors as therapeutic options for Sézary syndrome and other forms of CETL in veterinary patients.

## 1 Introduction

Cutaneous epitheliotropic T-cell lymphoma (CETL) is a malignant tumor that accounts for less than 1% of all canine skin tumors (1). It is defined by the infiltration of neoplastic T lymphocytes that exhibit an affinity for the epidermis and adnexal structures. This condition can be further categorized into three distinct subtypes: mycosis fungoides, pagetoid reticulosis, and Sézary syndrome.

Sézary syndrome is an uncommon subtype of CETL, characterized by the simultaneous presence of circulating neoplastic Sézary cells in the skin and peripheral blood (2). Sézary cells are lymphocytes with hyper-convoluted nuclei that appear cerebriform. Sézary syndrome has not been extensively studied in veterinary medicine. The syndrome was first reported in dogs in 1984 (3). It has the poorest prognosis of the three subtypes.

Common treatment options for CETL include lomustine and prednisone. The overall remission rate is approximately 80%, with partial remission occurring more frequently than complete remission (4). The median duration of remission was approximately 3 months. Recently, the off-label use of oclacitinib (Apoquel 5.4 mg®, Zoetis, Kalamazoo, Michigan, United States) has shown effective results in some cases of CETL (5, 6). However, little is known about the treatment for Sézary syndrome.

This report describes a dog with Sézary syndrome that was treated with oclacitinib, a Janus kinase (JAK) inhibitor.

## 2 Case description

A spayed female 10-year-old Yorkshire Terrier weighing 3.74 kg presented to the Veterinary Teaching Hospital of Chungbuk National University with severe, extensive cutaneous lesions. Additionally, the dog had systemic signs, including lethargy, polydipsia, and dysphagia, associated with multiple nodules affecting the mandible.

The patient exhibited pruritus with a Pruritus Visual Analog Scale (PVAS) score of 5. According to medical history, the dog had initially been treated at a local animal hospital, where topical chlorhexidine solution and a corticosteroid-containing ointment were prescribed based on gross visual examination without further diagnostic tests. However, no clinical improvement was observed. The dog lived indoors and was fed a commercial adult maintenance diet. Routine vaccinations were completed, and regular internal and external parasite control, including heartworm prevention, was maintained.

General physical examination revealed peripheral lymph node enlargement. The left prescapular lymph node measured 2 × 2 cm, whereas the bilateral popliteal lymph nodes measured < 1 cm. Except for generalized peripheral lymphadenopathy, physical skin examination revealed no significant abnormalities.

**Figure 1:**
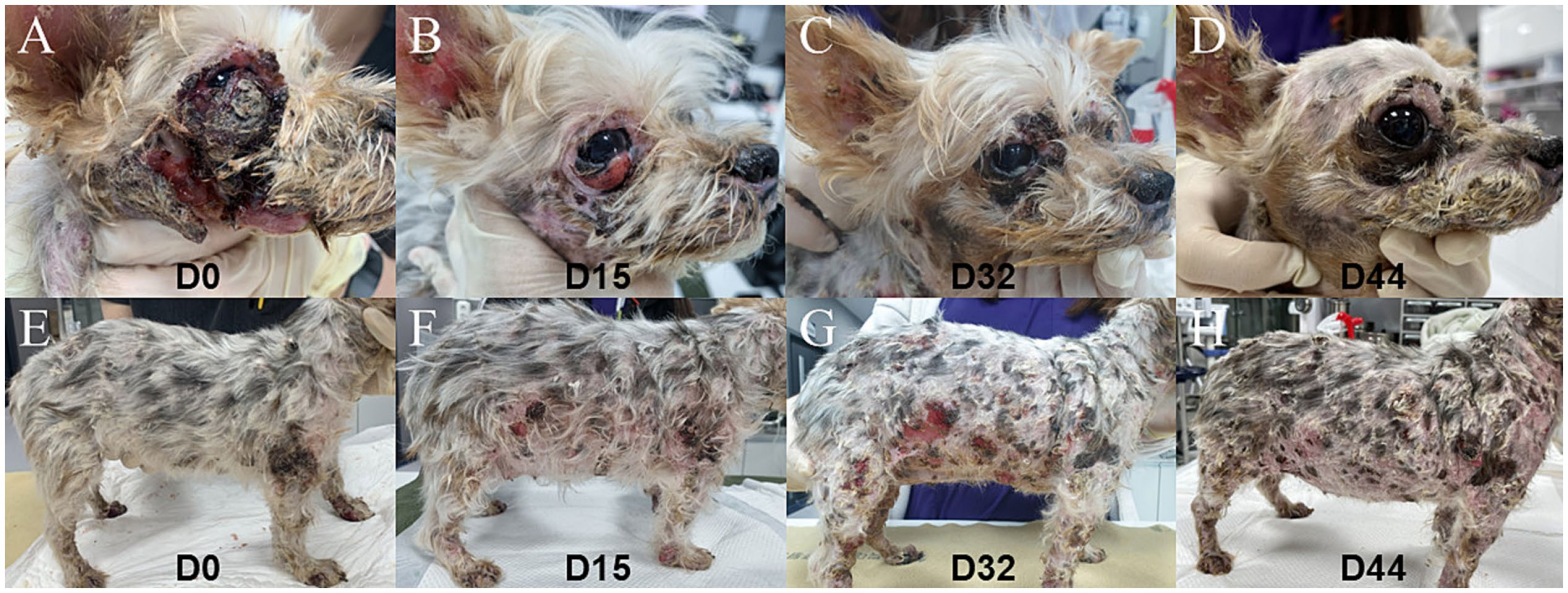
Gross lesion appearance on the face and trunk during treatment. **(A,E)** On D0, severe cutaneous crusts and ulcerations with hemorrhagic and seropurulent fluid were observed. **(B,F)** Marked improvement was observed on D15 of oclacitinib treatment (0.7 mg/kg, twice daily). **(C,G)** The facial and truncal lesion deteriorated on D32 of oclacitinib (0.7 mg/kg, twice daily) treatment. **(D,H)** On D44 of oclacitinib treatment (day 12 after dose escalation), the right lateral skin lesion showed improvement.

On visual examination, crusts and ulcerations with hemorrhagic and seropurulent fluid over the face and body that had progressively worsened over more than 3 months were found, as well as patchy alopecia on the lateral trunk and dorsal region, multiple palpable and firm cutaneous nodules on the mandible (Figures 1A,E).

## 3 Diagnostic assessment, therapeutic intervention, follow-up, and outcomes

**Figure 2:**
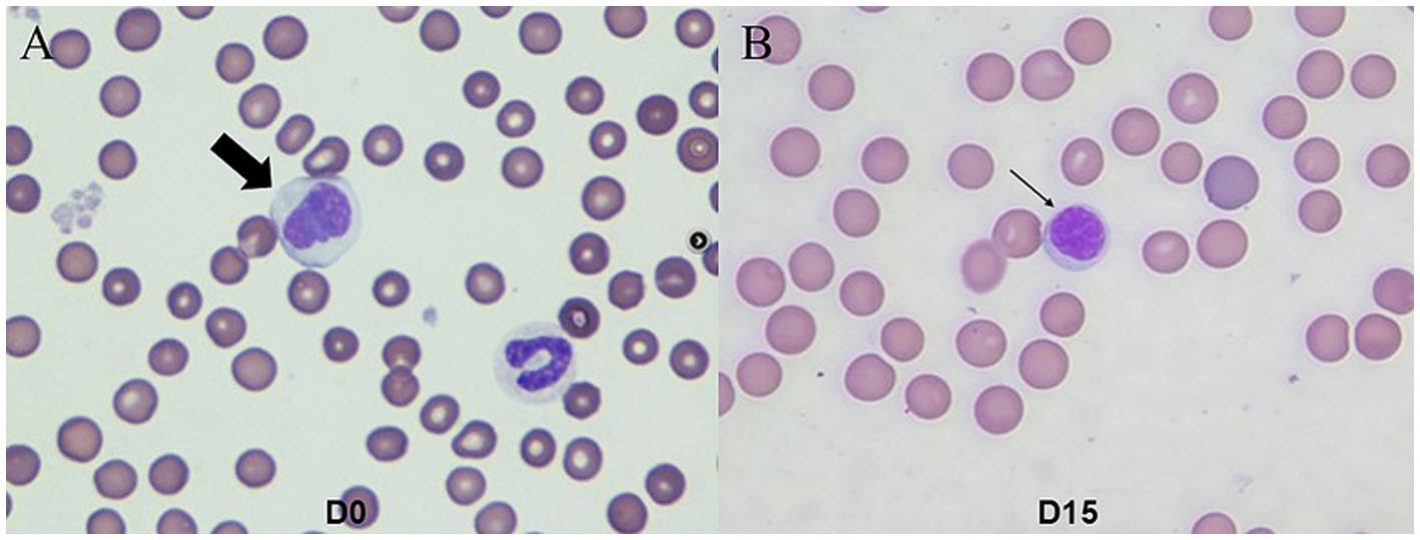
Peripheral blood smears in the dog with Sézary syndrome. **(A)** Most lymphocytes are observed to have a size approximately 1.5–2 times the diameter of erythrocytes on D0. The bold arrow indicates a Sézary cell exhibiting irregular cerebriform morphology with a coarse chromatin pattern. **(B)** The arrow indicates a normal lymphocyte with smooth, rounded nuclei on D15 of treatment. (Wright-Giemsa stain, ×1,000).

**Figure 3:**
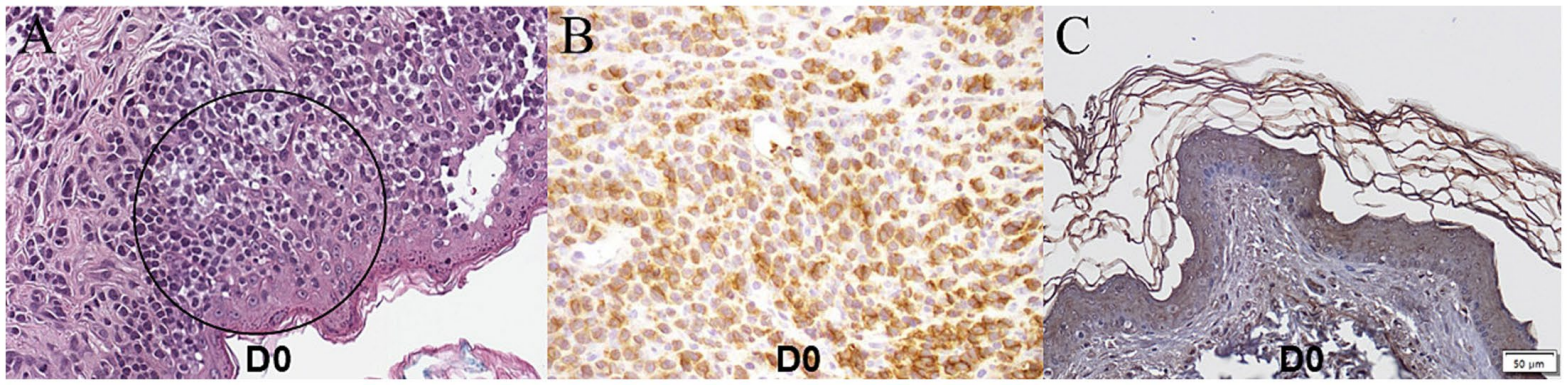
Histopathological and immunohistochemical examination of inguinal mass on D0. **(A)** Sheets of round cells supported by a pre-existing fibrovascular stroma are multifocally infiltrating and ulcerating the overlying epidermis and also diffusely infiltrating the dermis. **(B)** Immunohistochemical staining revealed CD3 positivity. **(C)** On immunohistochemical staining of JAK1, moderate and diffuse cytoplasmic and nuclear staining were observed.

Cytological evaluation of ulcerative skin lesions was performed using impression smears, which revealed numerous cocci (approximately 50 per oil-immersion field) and rods (>100 per oil-immersion field), as well as degenerated neutrophils. Active phagocytosis was observed. Fungal cultures were performed on hair and debris collected by brushing the skin with a sterile toothbrush, using Sabouraud dextrose agar and dermatophyte test medium, and both were negative, thereby excluding dermatophytosis. Skin scraping tests were performed to exclude demodicosis and other ectoparasitic diseases, with no parasites detected. Based on these findings, the dog was diagnosed with deep pyoderma. However, the presence of multiple cutaneous nodules suggested that the lesions were not solely attributable to bacterial infection. Given the dog’s age and the fact that cutaneous lesions developed prior to pruritus, canine atopic dermatitis was considered unlikely. Food allergy was also deemed improbable, as no changes in diet had occurred before disease onset. Hematological analysis, particularly complete blood count, revealed leukocytosis (24.36 × 10^3^/μL, reference interval [RI]; 5.05–16.96 × 10^3^/μL), neutrophilia (20.79 × 10^3^/μL, RI; 2.95–11.64 × 10^3^/μL), lymphocytopenia (0.22 × 10^3^/μL, RI; 1.05–5.10 × 10^3^/μL), monocytosis (3.09 × 10^3^/μL, RI; 0.16–1.12 × 10^3^/μL), normal basophil count (0.09 × 10^3^/μL, RI; 0–1.10 × 10^3^/μL), and normal eosinophil count (0.17 × 10^3^/μL, RI; 0.006–1.23 × 10^3^/μL). In serum biochemistry, increased CRP (70.39 mg/L, RI; 0–10 mg/L) was observed. Sézary cells were identified in peripheral blood smears (Figure 2A). On histopathological examination obtained by punch biopsy of the right inguinal region, sheets of round cells supported by a pre-existing fibrovascular stroma, multifocally infiltrating and ulcerating the overlying epidermis and diffusely infiltrating the dermis were observed (Figure 3A). Neoplastic cells displayed distinct borders, minimal eosinophilic cytoplasm, and round-to-oval nuclei with finely stippled chromatin and indistinct nucleoli. Mild anisocytosis and anisokaryosis were observed with a mitotic count of 18 per 10 high-power field (2.37 mm^2^). Immunohistochemical staining revealed CD3 positivity and CD79a negativity (Figure 3B), indicating that the round cells observed on histopathological examination were T-lymphocytes. Based on these findings, the patient was diagnosed with CETL, specifically of the Sézary syndrome subtype. A standardized clinical staging system for canine Sézary syndrome has not been established, unlike in humans, where the modified ISCL/EORTC TNMB classification is used for staging. However, based on the human criteria, the present case would correspond to stage T_3_N_x_M_0_B_1_ (7).

For the treatment of CETL, the owner declined aggressive chemotherapeutic agents such as lomustine because of concerns about potential adverse effects, including myelosuppression, hepatotoxicity, and gastrointestinal toxicity. Therefore, oclacitinib was selected as an alternative owing to its more favorable safety profile. On day (D) 0, treatment with oclacitinib 0.7 mg/kg, q12h and prednisolone 0.5 mg/kg, q24h (Solondo^®^, Yuhan, Seoul, Korea) was initiated. For bacterial infection management, 25 mg/kg of amoxicillin-clavulanate q12h (Zakutex Tab. 375 mg^®^, Theragen Etex, Ansan-si, Gyeonggi-do, Korea) and ciprofloxacin 30 mg/kg q24h (Prodin Tab. 250 mg^®^, CMG Pharmaceutical, Seongnam-si, Gyeonggi-do, Korea) were administered according to the results of the antibiotic susceptibility test. For topical management of infection, ketoconazole-chlorhexidine medicated shampoo (Ketochlor^®^, Virbac AH Inc., Fort Worth, Texas, United States) was prescribed because the fungal culture results were not yet confirmed as negative.

On D15 of treatment, a significant reduction in ulceration and crusting around the periocular and buccal regions was observed (Figures 1B,F). Hemorrhagic and seropurulent exudations were no longer evident particularly on the face, abdomen, neck, and forelimbs, although patchy alopecia and hyperpigmentation persisted (Supplementary Figure S1). Additionally, the nodules previously noted in the mandibular region had decreased in size. Cytological examination revealed a moderate presence of cocci, with approximately 30 organisms observed per oil-immersion field, showing improvement in skin infection. PVAS improved from 5 to 3, and the owner of the dog was satisfied with the clinical improvement observed. A complete blood count or serum biochemistry were not performed on that day. On blood smear examination, the lymphocyte nuclei showed no irregular contours, and no abnormalities were observed in the nuclear staining characteristics (Figure 2B). Sézary cells were no longer detected.

However, by D32, the lesions had significantly worsened, showing extensive ulceration, crusts, scales, and widespread erythema involving most areas of the body (Figures 1C,G). Compared to the day of diagnosis, complete blood count revealed more pronounced leukocytosis, increasing from 24.36 × 10^3^/μL to 35.29 × 10^3^/μL, and a stress leukogram was observed. Sézary cells were not identified in peripheral blood smears. In serum biochemistry, CRP was increased from 70.39 mg/L (on D0) to 254.49 mg/L. After the disease worsened on D32, the oclacitinib dosage was increased to 3 mg/kg twice daily, whereas the other treatments were maintained without changes.

Twelve days after dosage adjustment, on D44, marked healing of the severe ulcerative lesions on the lateral trunk was observed, accompanied by evident re-epithelialization of the affected skin (Figures 1D,H). Leukocytosis decreased from 35.29 × 10^3^/μL (on D32 when the dosage was increased) to 28.58 × 10^3^/μL, with a persistent stress leukogram. Sézary cells were not found in peripheral blood smears. Serum CRP decreased from 254.49 mg/L (on D32) to 8.45 mg/L, returning to the normal range. No adverse effects on hematologic examination were observed. However, on D63, cutaneous lesions similar to those observed on the first day of presentation recurred, showing marked aggravation throughout the trunk and the patient’s overall condition deteriorated. Leukocytosis increased again from 28.58 × 10^3^/μL on D44 to 42.20 × 10^3^/μL, showing a stress leukogram similar to previous patterns.

On D79 of treatment, the dog exhibited lethargy and reduced appetite, with a concurrent deterioration of the overall cutaneous condition. The complete blood count revealed marked lymphocytosis (10.13 × 10^3^/μL), and serum CRP increased from 8.45 mg/L (on D44) to 252.17 mg/L. Therefore, oclacitinib was deemed no longer effective and lomustine was included in the treatment regimen. However, cardiopulmonary arrest occurred twice the following day, and despite achieving return of spontaneous circulation, the patient was euthanized at the owner’s request. Cardiopulmonary arrest was presumed to have occurred due to severe dehydration and systemic deterioration, possibly associated with disease progression of CETL with systemic metastasis or systemic inflammatory response syndrome. Necropsy was not performed because the owner declined the procedure.

Because the dog eventually died, additional investigation was performed on the skin biopsy sample obtained on D0 to confirm whether oclacitinib had exerted a therapeutic effect at the molecular level. Immunohistochemical staining for JAK1, the target receptor of oclacitinib, was performed to evaluate the JAK1 intensity and distribution using JAK1 pY1022 polyclonal antibody (MyBioSource, San Diego, United States). Moderate and diffuse cytoplasmic and nuclear staining were observed in the preserved tissue sample from the biopsy (Figure 3C).

## 4 Discussion

This case report describes a canine patient diagnosed with Sézary syndrome who was managed with the off-label use of oclacitinib, a JAK inhibitor. Oclacitinib is a JAK inhibitor approved by the U. S. Food and Drug Administration in 2013 for the control of atopic dermatitis in dogs. This is the first approved JAK inhibitor for use in dogs (8). Treatment commenced at a dose of 0.7 mg/kg administered twice daily and was subsequently increased to 3 mg/kg twice daily. Both dosing regimens exhibited notable therapeutic effects. As the other treatments remained unchanged and only the oclacitinib dosage was increased, the subsequent improvement in the lesions clearly demonstrated the therapeutic efficacy of oclacitinib.

In human medicine, Sézary syndrome is characterized by erythroderma, generalized lymphadenopathy, and the presence of neoplastic T lymphocytes (Sézary cells) in the skin, lymph nodes, and peripheral blood (9). Because of its systemic nature, Sézary syndrome requires systemic therapy, which is often combined with skin-directed treatment. Systemic therapies include retinoids, interferon, and chemotherapeutic drugs like doxorubicin or gemcitabine. Skin-directed therapies include topical corticosteroids, phototherapy, and radiotherapy. In veterinary medicine, however, the diagnostic criteria and standardized treatment protocols for Sézary syndrome have not yet been established. To date, only six canine cases have been reported (2, 3, 10–13). Reported survival times were 0 days in two cases and 2, 14, 31, and 300 days in individual cases. The use of oclacitinib for canine CETL has been documented in two previous reports. In one case, a dog received oclacitinib at a dosage of 0.7 mg/kg twice daily, which led to approximately 80% improvement in ulcerative lesions by D23, followed by deterioration on D90 (5). In the second case, oclacitinib was initially administered at 1.6 mg/kg twice daily, resulting in therapeutic efficacy within 2 months, with deterioration observed on D91 of treatment (6). As the clinical response diminished, the dose was increased to 2.2 mg/kg twice daily, which led to only a partial therapeutic effect and did not demonstrate definitive therapeutic efficacy. The dosage was temporarily increased to 3 mg/kg twice daily and subsequently to 3.3 mg/kg twice daily. But the pruritus continued to worsen, and the dog was eventually euthanized. However, to date, there have been no reports on the use of oclacitinib in canine Sézary syndrome.

Immunohistochemical staining on skin samples in this case confirmed the expression of JAK1, the target receptor of oclacitinib, thereby providing a rationale for its therapeutic application. These findings suggest that oclacitinib and other JAK inhibitors are viable treatment options for canine cutaneous epitheliotropic T-cell lymphoma, particularly in cases of Sézary syndrome. It is crucial to emphasize that the therapeutic application described in this report constitutes off-label use of the drug. Although the mechanistic rationale supports its investigation, the safety profile and efficacy for oncological indications, especially at the elevated doses administered, have not been formally established. In the JAK/STAT signaling pathway, cytokines and growth factors initiate cell signaling and gene transcription by binding to transmembrane receptors and inducing conformational changes and JAK dimer phosphorylation. This activation leads to STAT protein dimerization, nuclear translocation, and the regulation of cytokine-specific gene transcription. JAK inhibitors disrupt this process by blocking JAK phosphorylation and nuclear signaling (14). In human studies, an association between the JAK/STAT signaling pathway and hematopoietic neoplasms or autoimmune diseases has been established. Particularly in cutaneous T-cell lymphoma (CTCL), the JAK/STAT pathway is mutated in up to 24% of CTCL samples. Most of these mutations affect the tyrosine kinase domain of JAK kinases, a hotspot for activating mutations described in multiple types of human cancer (15). Because frequent mutations affecting JAK/STAT downstream signaling were observed in samples from patients with CTCL, targeted inhibition of this pathway demonstrated that aberrant CTCL growth was controlled by this signaling.

Review studies of JAK inhibitors for CTCL treatment in humans have reported promising outcomes with acceptable side effects (14, 16). Few studies have investigated this topic in veterinary medicine. In 2024, a study investigated the inhibitory effects of four different JAK inhibitors (AZD1480, oclacitinib, INCB39110: itacitinib, CP-690550: tofacitinib) on cell growth and survival in canine B- and T-cell lymphoma cell lines (17). They concluded that AZD1480 exerted marked, dose-dependent inhibitory effects on all tested cell lines, leading to apoptosis and cell-cycle arrest, whereas oclacitinib demonstrated only modest inhibition at higher concentrations. In contrast, INCB39110 (itacitinib) and CP-690550 (tofacitinib) produced no significant antiproliferative effects. These findings indicate that selective inhibition of JAK2 signaling may represent a promising therapeutic strategy for canine lymphomas, warranting further investigation.

In the present case, oclacitinib dose adjustment to 3 mg/kg q12h was based on an *in vitro* finding showing that a 10 μM concentration, equivalent to the C_max_ achieved with oral administration of 3–4 mg/kg oclacitinib twice daily in dogs, exerted notable biological activity (18). Although the duration of treatment response was not prolonged, the survival time exceeded the previously reported survival time for canine Sézary syndrome. Given its mechanism of action targeting the JAK/STAT signaling pathway, the current evidence and its favorable side-effect profile compared to conventional chemotherapeutics, oclacitinib, and potentially other JAK inhibitors, may represent a viable therapeutic option for CETL in dogs, particularly in Sézary syndrome.

Adverse effects were not observed in this patient. According to previous reports, when oclacitinib is administered at the label dose and used according to label indications, adverse effects are uncommon, generally mild, and self-limiting. The most frequently reported signs include transient gastrointestinal disturbances such as vomiting, diarrhea, reduced appetite, or lethargy. Transient decreases in neutrophils, eosinophils, monocytes, and serum globulin, together with mild increases in cholesterol and lipase, may also occur. These hematologic and biochemical changes typically resolve within approximately 14 days and are not considered clinically significant (19).

This report has several limitations. First, as a single-case study (*n* = 1), the findings cannot be generalized to all dogs with CETL. Second, the concurrent administration of prednisolone and antibiotics may have confounded the interpretation of the clinical response to oclacitinib, as prednisolone exerts anti-inflammatory and immunomodulatory effects, while antibiotic therapy can indirectly alleviate inflammation by controlling secondary infection. Lastly, the clinical improvement was transient, lasting about 1 month after both dosing regimens, after which the lesions worsened again. Therefore, additional controlled clinical studies with larger case numbers and extended observation periods are warranted to further elucidate the therapeutic role of oclacitinib in this disease.

## Data Availability

The original contributions presented in the study are included in the article/Supplementary material, further inquiries can be directed to the corresponding author.
