# Supplementary material for: Case Report: Dose-dependent response to oclacitinib in a dog with Sézary syndrome
Source: Front Vet Sci. 2025 Dec 15;12:1645059. doi: 10.3389/fvets.2025.1645059 (PMC12745446; doi:10.3389/fvets.2025.1645059)
Supplement: Supplementary file 1 [file Data_Sheet_1.pdf]

Supplementary Material

1 Supplementary Data

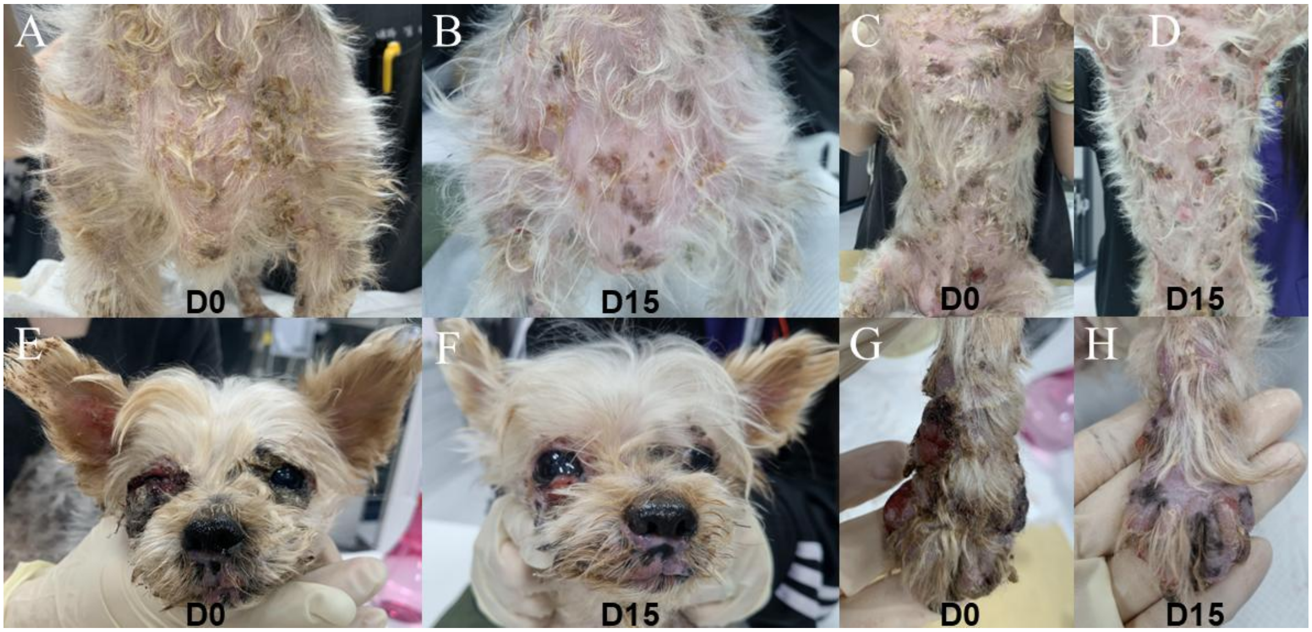

Supplementary Figure 1. Gross appearance of lesions on the neck, abdomen, face, and forefoot: (A, C, E, G) on D0 before treatment, and (B, D, F, H) on D15 after administration of oclacitinib (0.7 mg/kg, twice daily).
